# Characterization of Homeobox Genes Reveals Sophisticated Regionalization of the Central Nervous System in the European Cuttlefish *Sepia officinalis*

**DOI:** 10.1371/journal.pone.0109627

**Published:** 2014-10-06

**Authors:** Laura Focareta, Salvatore Sesso, Alison G. Cole

**Affiliations:** IRGS, Biogem, Ariano Irpino, Avellino, Italy; Laboratoire de Biologie du Développement de Villefranche-sur-Mer, France

## Abstract

Cephalopod mollusks possess a number of anatomical traits that often parallel vertebrates in morphological complexity, including a centralized nervous system with sophisticated cognitive functionality. Very little is known about the genetic mechanisms underlying patterning of the cephalopod embryo to arrive at this anatomical structure. Homeodomain (HD) genes are transcription factors that regulate transcription of downstream genes through DNA binding, and as such are integral parts of gene regulatory networks controlling the specification and patterning of body parts across lineages. We have used a degenerate primer strategy to isolate homeobox genes active during late-organogenesis from the European cuttlefish *Sepia officinalis*. With this approach we have isolated fourteen HD gene fragments and examine the expression profiles of five of these genes during late stage (E24-28) embryonic development (*Sof-Gbx, Sof-Hox3, Sof-Arx, Sof-Lhx3/4, Sof-Vsx*). All five genes are expressed within the developing central nervous system in spatially restricted and largely non-overlapping domains. Our data provide a first glimpse into the diversity of HD genes in one of the largest, yet least studied, metazoan clades and illustrate how HD gene expression patterns reflect the functional partitioning of the cephalopod brain.

## Introduction

Homeodomain (HD) transcription factors are important regulators of developmental patterning across animal lineages, defined by the presence of a 60aa DNA binding homeodomain. The antennapedia (ANTP) class of homeodomain transcription factors is the largest group of animal homeodomain (HD) genes (see http://homeodb.zoo.ox.ac.uk/ [1] [2]). The ANTP-class HOX sub-group genes in particular have been widely investigated since the discovery of their conserved role in axial patterning of body plans throughout metazoan lineages [3]. However, there are many other families of HD proteins which have been less well studied in a comparative, evolutionary context. The second largest homeobox gene group is the paired-class (PRD), as defined by the presence of the PRD-homeobox [4]. Where they have been studied, PRD-class genes show expression within developing nervous systems, as well as involvement in axial patterning [5] [6] [7]. The LIM-class of HD transcription factors, which includes proteins with a zinc-finger (LIM) domains, have been well studied for their role in neural specification from flies to vertebrates [7]–[11]. Thus, it is clear that a number HD transcription factors play a pivotal role in the generation of highly organized central nervous systems.

**Figure 1:**
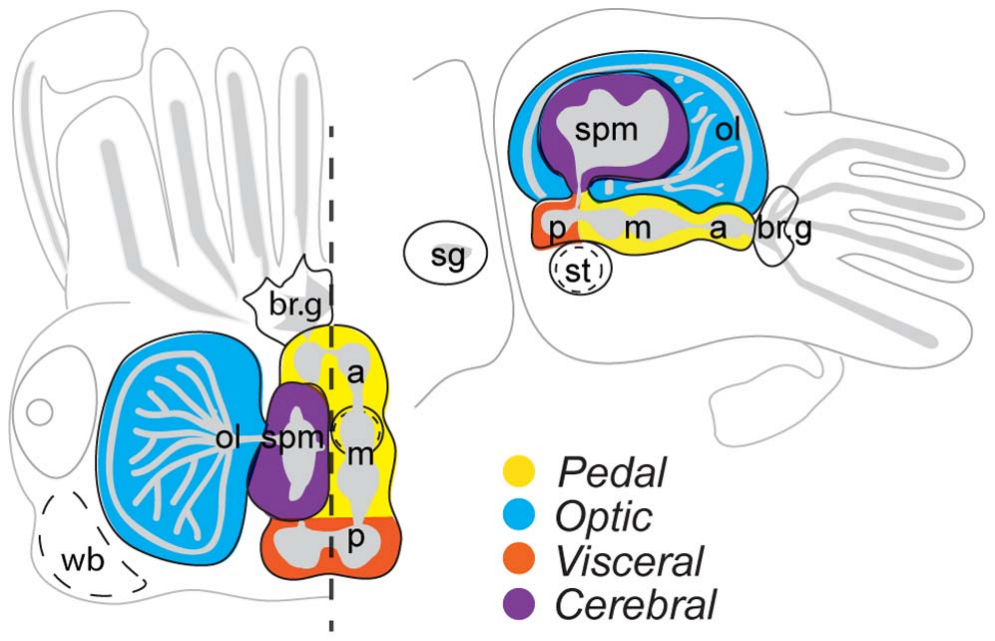
Schematic diagram of the cuttlefish CNS. The molluscan ganglia origin of each principle lobe of the central nervous system (CNS) is color coded. On the left the head is shown from a dorsal view, with the right supraesophageal mass (SPM) removed at the mid-line (dashed line). The CNS is represented in lateral view on the right. The grey neuropil structure is approximate and not meant to represent the actual internal structure of the organ. a: anterior subesophageal mass; br.g: branchial ganglia; m: middle subesophageal mass; ol: optic lobe; st: statocyst; p: posterior subesophageal mass; sg: stellate ganglia; spm: supraesophageal mass; wb: white bodies.

Contrary to the situation found in arthropods, nematodes, echinoderms, and chordates, where the genetic mechanisms that underlie embryonic development have been well studied, little is known about the genetic control of development in cephalopod mollusks. Transcriptome analysis of the adult central nervous system from an octopus reveals approximately 3% of the active transcriptome is involved in transcription factor activity [12]. Given the widespread use of HD proteins during neural specification in other metazoan lineages, we investigate HD proteins active during maturation of the central nervous system of the cephalopod mollusk *Sepia officinalis*. Development of the European cuttlefish, *Sepia officinalis*, is typical of most cephalopod molluscs with 30 morphologically described embryonic stages (E1–30: [13] [14]). Embryonic stages can be grouped into five developmental phases corresponding to cleavage and gastrulation (stages E1–15), primordial organ placode formation (disc phase: stages E15–20; [15]), organogenesis and differentiation (stage E18–27; [14]), tissue maturation (stage E28–29; [14]) and hatching (stage E30). During the straightening phase of embryonic development, when the embryonic disc elevates from the yolk (Stages E21–22; [15]), paired neural placodes are concentrated and fuse medially to form the lobes of the adult CNS that surround the eosophagus within the head. The subeasophageal mass (SM) is composed from the visceral (posterior SM) and pedal (medial and anterior SM) ganglia placodes, and the supraesophageal mass (SPM) derives from the cerebral ganglia placodes, with optic lobes located laterally behind each eye (Figure 1; [16] [17]). Analysis of ELAV expression, a marker of postmitotic neural cells, has revealed asynchronous maturation of each lobe of the CNS –the pSM is the first lobe to contain mature neurons as early as stages E15/16, followed by the mSM and aSM at stage E20, whereas the SPM is the last to contain maturing neurons at stage E22 [17]. We recover homeodomain proteins of the ANTP- PRD- and LIM- classes, and find all to be expressed within the maturing central nervous system in largely un-overlapping domains during late stage embryogenesis (E24–28).

## Materials and Methods

### Ethics statement

The use of embryonic cephalopod material for research is currently not legislated in Europe and thus no permits were required for this work. Nonetheless, adult cephalopods are now included in European legislation in order to avoid perceived pain and suffering that may be associated with their highly functioning central nervous system. See Moltschaniwskyj *et al.* [18] and Fiorito *et al.* [19] for discussion on ethical issues involving cephalopods in research. All animal material was treated with the most humane care possible – embryos used in this study were lethally anesthetized in ethanol prior to sacrifice at stages prior to yolk absorption.

### Animals

Embryonic material used in this study derive from fertilized eggs naturally deposited on fishing nets from the seawaters near Ancona Italy (April 2010, 2012). Deposited eggs that were normally cleaned off the nets and dumped in the sea were collected by licensed fishermen and brought to shore. The authors transported the egg capsules to the laboratory where they were kept in 20% artificial sea water (ASW: TETRA Marine SeaSalt) and 80% natural sea water (Gulf of Naples) with aeration at room temperature in a closed salt water aquarium. Embryos prior to yolk absorption were isolated from egg envelops, anesthetised in 1% EtOH, and staged according to Cole and Hall [14]. Embryos destined for RNA extraction were placed in TRIzol Reagent (Invitrogen, 15596-018) and stored at -80°C until use; embryos destined for *in situ* hybridization were fixed in 4% paraformaldehyde (PFA) overnight, washed in PBS and incubated in 30% sucrose in PBS overnight prior to embedding in optimum cutting temperature (OCT) compound (Killik – BioOptica, 05-9801), and stored at −80°C until sectioning.

### Gene cloning

Staged embryos were homogenized in Trizol with a tissue lyser (Qiagen), and RNA extraction was performed according to manufacturer's recommendation. An mRNA isolation kit (ROCHE) was used for the isolation of poly(A+) RNA, following the manufacturer's instructions. Purified RNA was stored at −80°C until use. cDNA was retro-transcribed from equal quantities of purified poly(A) or total RNA extractions from embryos of stage E24-E30, using the SuperSript First-strand Synthesis System (Invitrogen) according to the manufacturer's instructions.

Two pairs of degenerate primers, designed to amplify 96 bp between the first and third conserved helix of the homeodomain (HD), were used to amplify ANTP- (ANTfw: CGGATCCYTIGARYTIGARAARCART; ANTrv: GGAATTCATICKICKRTTYTCRAACCAIAT) and PRD- (PRDfw: CAGCTSGARGARCTGGAG; PRDrv: GCBCKNCGRTTYTGRAACC) class HD fragments. Degenerate PCR was performed in a total volume of 20 µl containing 10 ng of mixed-stage cDNA (stages E24-E30) and 2 µM of each primer for 35 cycles using the following thermal profile: 30 s at 94°C, 30 s ramping from 40–55°C and 45 s at 72°C followed by a final elongation step of 10 m at 72°C. The resultant 120 bp amplicon was cloned into a pCRII-TOPO vector (Invitrogen, K4600-01), and 50 positive colonies were sequenced. Sequenced gene fragments were identified according to BLAST similarity and orthology assignment of the genes was determined following phylogenetic analysis of the conserved HD (Figure S1). Homeodomain sequences were downloaded from the homeoDB (http://homeodb.cbi.pku.edu.cn/ [1] [2]) and complemented with Mollusk and Annelid sequences found in GenBank. Amino acids 18–31 of the conserved 60aa HD were aligned (Supporting Information S1a) and imported into Clustal Phylogeny, using the neighbour-joining algorithm with the “exclude gaps” option activated (http://www.ebi.ac.uk/Tools/phylogeny/clustalw2_phylogeny/). Newly described sequences for HD fragments that were not successfully extended, and thus too short for GenBank submission are found in the supplementary material (Supporting Information S1b).

### RACE PCR

cDNA derived from pooled RNA extractions, covering stages E24–E30 as described above, was used as template in all RACE PCR amplifications. The partial coding sequence for seven of the fourteen Sof-HD gene fragments were extended towards the 5 prime end using a SMARTer (Switching Mechanism At 5′ end of RNA Transcript) RACE cDNA amplification kit (CLONTECH) according to the manufacturer's protocol, using two rounds of gene specific amplification using the following primers: SoHox1Rv1 – CGGGCCCGTGTCAGGTATTTATTGA; SoHox3Rv1 – TCTGTCGTTCGGAGAGGTTCAGCAG; SoGbxRv1 – CGCTAAGCTTCAGGTTGTGGGCAAT; PRD-class: SoDrgxRv1 - ATCCGGATAATGCGTTTGAGCGAAG; SoVsxRv1 – CCTCCGGCAGGTCTGTCTTCAGAG; SoArxRv1 - CGAGCTTCCGTAAGATCAATTCTCAAAGC; SoLhx3/4Rv1 – CCACTCTCATGTCAAGTCCGGTTTCA. *Sof-Gbx, Sof-Vsx* and *Sof-xLox* were extended towards the 3 prime end using a 5′/3′ RACE Kit, 2^nd^ Generation (03 353 621 001 - ROCHE) following the manufacturer's protocol using two rounds of amplification with the following gene specific primers: SoXLoxFw1 – CGAAATCTCATGCTCACCAA and SoXLoxFw2 – TACACGCGGTCAACTTCTTG; SoVsxFw1 – AGGCCCATTACCCGGATGTTTATGC and SoVsxFw2 – CCCAGCAAGACCAGTTGAGCCTTTT; SoGbxFw1 – TCTTTGACCGAGCGTTCGCAGATT.

### Reverse transcription polymerase chain reaction (RT-PCR) analysis

PCR amplification was performed using 5 ng of template cDNA, derived from staged embryo total RNA extractions, for 30 cycles (Sof-HD genes) or 20 cycles (RPS16 internal control) with 55°C as the annealing temperature. 0.5 µM of each primer pair were used as follows: SoXLoxFw1 and SoXLoxRv1- CGGTTTTGGAACCAAATTTTTA (304 bp); SoGbxFw2 - TCACAACACGTGGAACATGA and SoGbxRv2 - GAACGCTCGGTCAAAGAAAG (347 bp); SoHox1Fw1 - GGCGGGTATCGTATGCAC and SoHox1Rv2 - CGCTCCTTGGGAATTTGAG (212 bp); SoHox3Fw1 - GTGCCTACTCGAACCCATGT and SoHox3Rv2 - CGACAAGTTGAGCGCTTGTA (314 bp); SoArxFw1 - AACGTAAGCATTGCAGAGGAC and SoArxRv2 - CCTTCTCCAGTTCCTCCAACT (457 bp); SoDrgxFw1 - TTCACTGGCCACCAACATTA and SoDrgxRv2 - TCAAGCTGCTGGAGAGTGAA (169 bp); SoVsxFw3 - ATCACCGTTCGCTATTCAGG and SoVsxRv2 - CTTTGTCATCAACGCCCTCT (259 bp); SoLhx3/4Fw1 - TTAACACCGGGGATGAGTTC and SoLhx3/4Rv2 - GGTTTCAACACTCAGCTGCTC (215 bp); RPS16Fw1 - GGTTTGACGAAGGTTTACCTG and RPS16Rv1 - CGCTGTTATCCCTATGGTAAC (264 bp).

### qPCR

To analyze the temporal expression profile of Sof-HD genes, qPCR experiments were carried out as described below, using the following gene specific primers: SoArxFw2 - CGTCCACTGGCAACGGAGAATATCA and SoArxRv2 (153 bp); SoDrgxFw2 – CGGTTACCCGACTTGGTATG and SoDrgxRv2 - AGAGTGAAAGTAGTGCGGTTCC (133 bp); SoHox3Fw1 and SoHox3Rv3 - GTTGCTTCAAGAGGGACTGG (141 bp); SoLhx3/4Fw3 - ATATGACTTGGACGGCGGTA and SoLhx3/4Rv3 - TCATGTCAAGTCCGGTTTCA (146 bp); SoVsxFw4 – CCGGACACAGTACTCCGTTT and SoVsxRv2 (134 bp); SoXLoxFw2 – GCGAATAATAATGCCGGAAA and SoXLoxRv2 - CCATCCCTGTTGCTTGAACT (134 bp); SoGbxFw3 - TGGCAAAACAGACGACTTTG and SoGbxRv2 - TCGCTAAGCTTCAGGTTGTG (171 bp); SoHox1Fw2 - ACGAGACGCACACTGATCTTT and SoHox1Rv3 TTGCCATGTCCACCGTTT (145 bp); RPS16Fw2 - AAAAAGAAGTTTTAGTTGGGGTGA and RPS16Rv1 (129 bp).

Total RNA extracts from 3 same-staged individuals was pooled and cDNA was retro-transcribed using the SuperSript First-strand Synthesis System (Invitrogen) according to the manufacturer's instructions. PCR amplifications were performed using 5 ng of cDNA as template with 0.3 µM primer and 1X FastStart Universal SYBR Green Master (ROX); each assay included a no-template control for each primer pair and were carried out in duplicate. All genes were included in at least three independent assays. Data were normalized to RPS16 levels as an endogenous reference for each assay run, the expression level of which remains relatively constant in all the developmental stages examined (RPS16_fw2 AAAAAGAAGTTTTAGTTGGGGTGA and RPS16_rv as above (158 bp) [20]. To visualize expression profiles of individual genes, fold-change in gene expression levels were calculated relative to the sample with the lowest expression level using the 2^−ΔΔCt^ method; standard errors in fold-change values were calculated with respect to standard deviations in the raw data according to [21].

### *In situ* hybridization

RNA probes corresponding to 5′ RACE products were transcribed with the Sp6-T7 kit from Roche as recommended by the manufacturer: DIG RNA Labelling Kit (Roche 11 175 025 910) was used for the *in vitro* transcription: *Sof-Arx1* (533 bp); *Sof-Gbx* (571 bp)*; Sof-Lhx3/4* (234 bp)*; Sof-Hox3* (402 bp)*; Sof-Vsx* (390 bp). 10 µm frozen sections were cut with a cryostat (MICROM HM500) under RNAase-free conditions, and mounted on SUPERFROST PLUS slides (Menzel-Gläser, J1800AMNZ). These prepared slides were processed for *in situ* hybridization following the protocol of Little *et al.* [22] with the following modification: Proteinase K was used at a concentration of 1 µg/ml in PBS. Probe localization was visualized using incubation with BM Purple (Roche, 11442074001) until the signal reached satisfactory intensity and mounted with Glycergel (Dako - C0563). Mounted slides were digitally imaged with a Zeiss Axioplan2 microscope using the Axiovision REL.4.8 software. All images presented in this work were processed in Adobe Photoshop CS5 using a High Pass filter (250 pixel radius) to remove lighting artefacts introduced by the imaging system, and subsequently adjusted for Brightness and Contrast (see Figure S2 for an example of a pre- and post-processed image). Schematic illustrations of expression domains were generated with Adobe Illustrator CS5.1 based upon analysis of serially sectioned material and relevant data from the literature.

## Results

We used degenerate primers specific for the conserved portion of the paired-class (PRD) and antennapedia-class (ANTP) homeodomain to identify homeodomain (HD) proteins expressed during late-stage organogenesis in the cuttlefish *Sepia officinalis*. Using this strategy we identified 28 amino acids that correspond to amino acids 19–46 of the conserved 60aa homeodomain for four PRD-class HD proteins: dorsal root ganglia homeobox (*Sof-Drgx*), visual system homeobox (*Sof-Vsx*), prophet of Pit1 homeobox (*Sof-Propx*), and the aristaless-like homeobox (*Sof-Arx*); and nine ANTP-class HD proteins: gastrulation brain homeobox (*Sof-Gbx*: new), distalless-like homeobox (*Sof-Dlx*: new), paralogy group labial/Hox1 (*Sof-Hox1*: identical to CAI77461.1), paralogy group Hox5/Scr (*Sof-Scr*: identical to CAI77463.1), paralogy group Hox3 (*Sof-Hox3*: identical to CAI77462.1), paralogy group ubx/Lox2/Hox7-8 (*Sof-Lox2*: new), paralogy group Lox4/Hox8 (*Sof-Lox4*: identical to CAI77464.1), paralogy group ant/Lox5//Hox6/7 (*Sof-Lox5*: new), and the ParaHox protein xLox/Pdx (*Sof-xLox*: new). We also recovered one LIM-class homeodomain of the LHx3/4 group (*Sof-Lhx3*/4: new). Neighbour-joining analysis based upon alignment of aa19–46 of the homeodomain is illustrated in Figure S1. The *Sepia* HD fragments group with other molluscan sequences where available, and are clearly identifiable as orthologs of the identified gene families.

Of these HD gene fragments, seven sequences were successfully extended towards the 5prime end (*Sof-Arx; Sof-Drgx, Sof-Vsx, Sof-Gbx, Sof-Lhx3/4, Sof-Hox1, and Sof-Hox3*) and two towards the three prime end (*Sof-Gbx,*and *Sof-xLox*) using RACE amplification with gene specific primers. Analysis of the coding sequence reveals no identifiable conservation outside of the HD for *Sof-Arx* (data not shown). The recovered *Sof-Vsx* sequence shows similarity in the 5′ end with the *Crassostrea Vsx2* sequence (EKC18872.1; data not shown), which suggests that this gene extends beyond the sequence recovered here. In contrast, 5′ extension of *Drgx, Gbx, Lhx3/4, Hox1, xLox and Hox3* gene sequences reveals a high degree of protein sequence conservation with available molluscan sequences (Table 1). Interestingly, the *Sof-xLox* protein has two isoforms, the longer of the two containing an additional 28 amino acids upstream of the HD (GenBank accession n. KJ467080), in the same position as a similarly sized exon identified from genomic DNA in the sea star [23].

**Table 1:** Details of recovered HD gene sequences from the European cuttlefish *Sepia officinalis.*

Gene name	Recovered Sequence length (nt)	GenBank accession number	Gene portion covered	Best BLAST Hit*	% identity*
*Sof-Arx*	653	KJ467073	5 prime + HD	*Capitella* (ELT87482.1)	96% (aa167–217)
*Sof-Drgx*	292	KJ467077	5 prime + HD	*Lymnea*_Drgx (AGC24174.1)	74%
*Sof-Gbx*	571	KJ467079	Entire coding	*Crassostrea*_Gbx (EKC23204.1)	89%
*Sof-Lhx3/4*	244	KJ467076	5 prime + HD	*Lymnea*-Lhx3/4 (AGC24171.1);	91%
*Sof-Hox1*	515	KJ467075	5 prime + HD	*Euprymna*_labial AY330184.1	90%
*Sof-Hox3*	418	KJ467074	5 prime + HD	*Euprymna*_Hox3 (AAR16188.1);	91%
*Sof-Vsx*	550	KJ467078	Partial 5 prime + HD	*Crassostrea*-Vsx2 (EKC18872.1)	49%
*Sof-xLox*	1052	KJ467080, KJ467081	Hexapeptide, HD+3 prime	*Euprymna*_xLox (ABD16192.1)	90% (with short isoform: KJ467081)

*derived from the NCBI “nblastn” algorithm (http://blast.ncbi.nlm.nih.gov/Blast.cgi).

**Figure 2:**
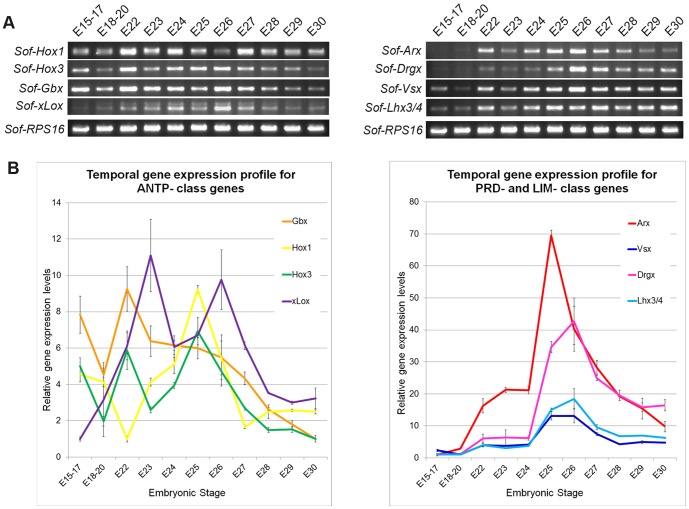
Temporal gene expression profiles of homeodomain genes. A) Reverse transcription polymerase chain reaction (RT-PCR) analysis of ANTP-class genes (left) and PRD- and LIM-class genes (right) amplified for 30 PRC cycles. The ribosomal protein control (Sof-RPS16) shows a saturated profile after 20 cycles, whereas all developmental transcription factors show differential peaks of expression throughout embryogenesis. B) Real time quantitative reverse transcription polymerase chain reaction (qPCR) analysis of ANTP-class genes (left) and PRD- and LIM-class genes (right). Ct data for each gene was normalized to the internal control (Sof-RPS16), and represented relative to its lowest levels of gene expression present in the assay. The ANTP-class genes show oscillating expression profiles throughout embryogenesis, whereas the PRD- and LIM-class genes show very similar expression profiles with an expression peak between stages E25–26.

We proceeded to investigate the temporal expression dynamics for these eight HD transcription factors from stages E15–30. *Sof-Arx, Sof-Drgx,* and *Sof-xLox* are nearly undetectable with RT-PCR prior to stage E22, whereas all other genes are detectable with this highly sensitive method in all stages investigated (Figure 2A); qPCR analysis confirms this expression profile for all of these eight genes (Figure 2B) in three independent assays. Overall, the relative gene expression profiles highlight the oscillatory nature of ANTP-class genes with respect to PRD- and LIM- gene families, and show that ANTP-family genes appear to be active earlier in embryonic development with respect to the PRD- family genes (Figure 2B). All genes examined show the highest peak of expression between embryonic stages E24–27. We investigated the spatial expression patterns of five of these genes in this developmental window: two ANTP-class genes *Sof-Hox3* and *Sof-Gbx*, two PRD-class genes *Sof-Arx*, and *Sof-Vsx*, and one LIM-class gene *Sof-Lhx3/4*.

**Figure 3:**
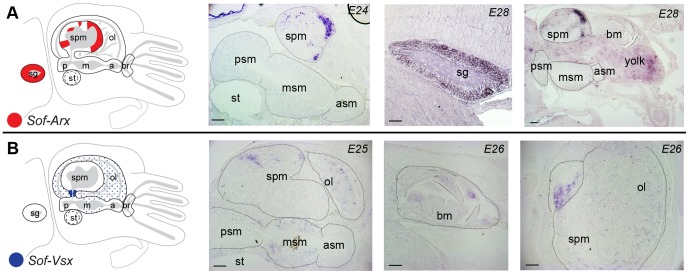
Spatial gene expression profiles of PRD-class homeodomain genes. The first panel of all figures is a schematic diagram as in figure 1, summarizing gene expression domains reconstructed from analysis of serial sections of embryos from stages E24–28. The age of embryo shown is found in the upper right corner of each image. A) *Sof-Arx*. From left to right: Medial section through the head illustrating expression within the supraesophageal mass; Gene expression within the stellate ganglion; Medial section through the head illustrating retained expression within the supraesophageal mass. B) *Sof-Vsx*. From left to right: Medial section through the head illustrating expression within cells of the optic lobes, the supraesophageal mass, and the middle subesophageal mass; section through the buccal mass illustrating signal associated with the mesenchyme of the developing beak; medio-lateral section through the head demonstrating gene expression in cells scattered throughout the optic lobes, and within the supraesophageal mass. Abbreviations - asm: anterior subesophageal mass; bm: buccal mass; msm: middle subaosophageal mass; psm: posterior subesophageal mass; st: statocyst; sg: stellate ganglion; spm: supraesophageal mass.

Spatial analysis of expression patterns on frozen sections shows that each of the five HD genes are expressed within the developing nervous system in a pattern that is largely invariable throughout this phase of embryonic development. In particular, *Sof-Arx* is homogeneously expressed within the stellate ganglia (SG) and in the anterior most portion of the supraesophageal mass (SPM) corresponding to the adult frontal lobes, with smaller more restricted domains more posteriorly in the vertical lobe (Figure 3A). This expression pattern is present at stage E24, and is maintained throughout the period of embryogenesis examined here (up until stage E28). *Sof-Vsx* is expressed in individual cells scattered throughout the optic lobes (OL; Figure 3B), as well as in a concentrated expression domain in the dorsal basal lobes, at the junction between the OL and the SPM, from stages E24-28 (Figure 3B). Between stages E25–26 expression is also detected in a population of cells scattered throughout the middle subesophageal mass (mSM; Figure 3B), whose expression may contribute to the observed peak in relative embryonic expression seen at this stage. *Sof-Vsx* is also expressed within the mesenchymal tissues of the developing beak in the buccal mass from stages E24–27 (Figure 3B).

**Figure 4:**
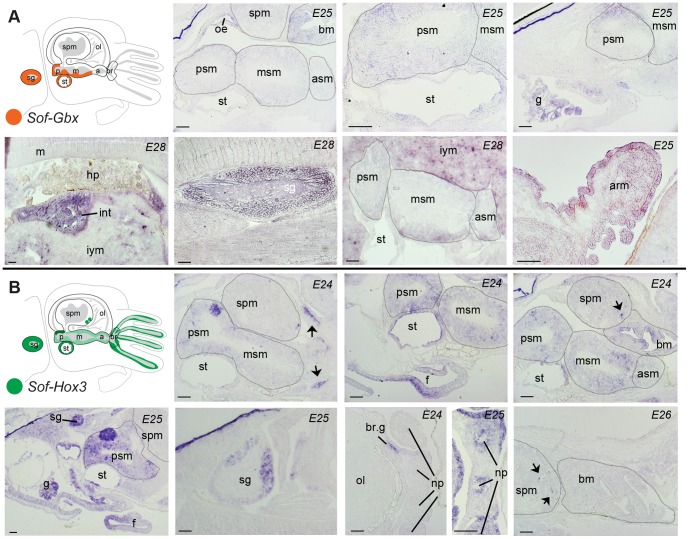
Spatial gene expression profiles of ANTP-class homeodomain genes. Figure layout as in Figure 3. A) *Sof-Gbx*. Clockwise from upper left: Medial section through the head showing signal within the buccal mass, oesophagus, and posterior and middle subesophageal masses; Higher magnification from the same embryos showing gene signal within the posterior subesophageal mass and statocyst; Media-lateral section through the mantle cavity highlighting expression in the gills and posterior subesophageal mass; Expression is detected in the arm epithelium; Medial section of the subesophageal mass, signal is evident in the ventral-most regions; Staining of the stellate ganglion; Medial section through the mantle cavity highlighting gene expression in the intestinal apparatus. B) *Sof-Hox3*. Clockwise from upper left panel; Medial section through the head showing extensive staining within the posterior and middle subesophageal masses, highlighting a domain of concentrated gene expression posteriorly. Signal is also present at the level of the brachial ganglia (arrows); Section showing medial staining within the funnel; Section shows staining in the beak epithelium in the buccal mass and a small population of 2–4 cells (arrow) in the anterior-most portion of the supraesophageal mass; Buccal mass staining is no longer present by stage E26, whereas the supraesophageal mass cells retain expression (arrows); Section through the base of the arms in a stage E25 embryo shows staining within the arm epithelium and the nerve cord surrounding the neuropil of the arms (np); Similar staining within the arms is not present in earlier stage E24 embryos, while expression in the brachial ganglia primordial is present; Abdominal section showing staining in the stellate ganglia; Lower magnification image showing staining in gills, stellate ganglia, subesophageal mass, and ventral-most portion of the funnel tube. Abbreviations - br.g: brachial ganglia; g: gills; hp: hepatopancreas; int: intestine; iym: inner yolk mass; m: mantle; np: neuropil; oe: oesophagus; all others as in Figure 3.

The ANTP-class genes investigated here are largely restricted to the subesophageal mass (SM). *Sof-Gbx* shows the most expansive expression domains of the genes investigated; it is expressed in the SG, on the ventral floor of the mSM and statocyst epithelium, and throughout the pSM from stages E24 through E28 (Figure 4A). *Sof-Gbx* also shows expression in tissues outside of the nervous system from stages E24–28, in particular within digestive system, mesenchyme of the buccal mass, the gills, and the arm epithelium (Figure 4A). *Sof-Hox3* expression is detectable at the level of the nervous system from stage E24, found within the SG, the brachial ganglia at the base of the arms (Figure 4B), and expression within the nerve cord of the arms appears at stage E25 (Figure 4B). Within the CNS, *Sof-Hox3* is expressed in cells throughout the SM, with a concentrated expression domain in the dorsal-medial portion of the pSM (Figure 4B). A few cells in the anterior-most portion of the SPM (adult inferior frontal lobe) also express *Sof-Hox3*, but no expression is detected in the OL. Expression is also found in non-neural tissues from stage E24, including the gills, the epithelium of the beak, the medial portion of the external wall of the funnel tube, the white bodies, and the epithelium of the arms (Figure 4B). These expression patterns remain stable though stage E27, with the following exceptions: expression in the epithelium of the beak and the gills disappears by stage E26, and is no longer detectable within the funnel by stage E27.

**Figure 5:**
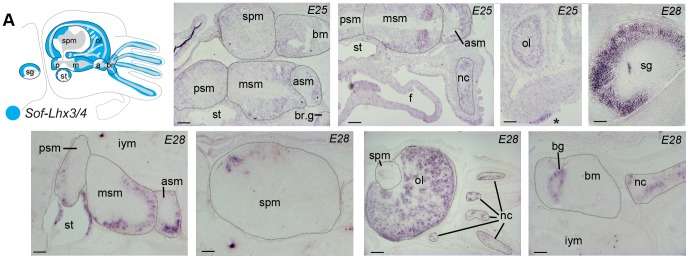
Spatial gene expression profiles of *Sof-Lhx3/4*. Figure layout as in Figure 4. Clockwise from upper left: Medial section through the head detecting moderate expression levels within all lobes of the CNS and the posterior mesenchyme of the buccal mass; A more lateral section of the same embryo, highlighting the strong expression domain within the middle and anterior subesophageal masses, and expression within the nerve cords of the arms; Lateral section showing expression within the optic lobes and the presumptive olfactory pit epithelium (*); Expression within the stellate ganglion is absent in the ventral-anterior most portion; Medial section at the level of the buccal mass showing expression within the buccal ganglion and neural cord of the arms; Section at the level of the optic lobes demonstrating expression throughout. Expression is also evident in the nerve cords of the arms but absent in the dorsal-lateral portion of the supraesophageal mass; Medial section through the supraesophageal mass showing expression domain in the dorsal-anterior most portion; Medial section through the subesophageal mass illustrating expression domains within the anterior-most portion of the anterior and middle masses, as well as the posterior-most portion of the posterior mass and underlying statocyst. Abbreviations: bg: buccal ganglia; nc: nerve cord; all others as in Figures 3 and 4.

The single LIM-class gene identified here, *Sof-Lhx3/4*, is expressed in all lobes of the CNS in spatially restricted domains in stages E24-28. It is detected in a population of cells scattered throughout the OL, in a small domain in the dorsal-posterior SPM (vertical lobe), in the posterior-most pSM, in the mid-anterior mSM, the anterior aSM, the brachial ganglia and nerve cord of the arms, and within the SG where the cell population is restricted to the dorsal portion of the ganglia, absent from the interior-most ventral zones (Figure 5). *Sof-Lhx3/4* is also expressed within the statocyst epithelium, the buccal ganglion, and the sensory epithelium of the presumptive olfactory pit (Figure 5). At stages E25/E26 expression is found within the arm epithelium; this expression domain was not detected in any other stage investigated here.

## Discussion

**Figure 6:**
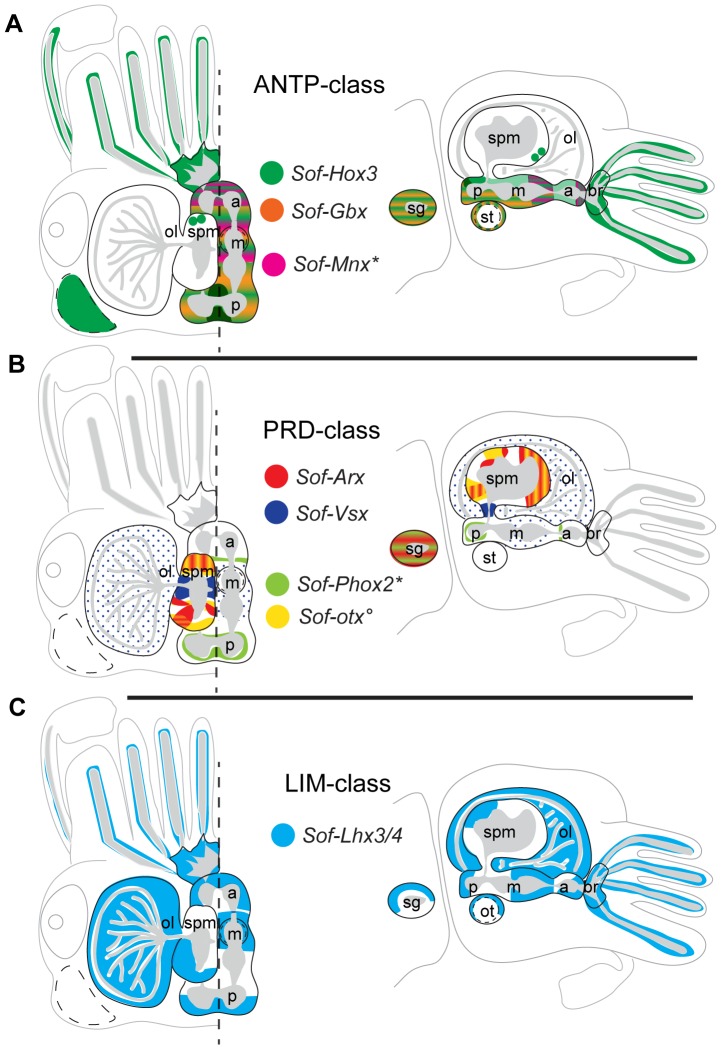
Schematic representation of HD gene expression domains in the cuttlefish CNS. Expression territories are extrapolated from analysis of serially sectioned embryos from stages E24–28 (this paper) and previously published data^*^ °. Dorsal view of the head is shown on the left, and lateral view on the right as in Figure 1. Territories with potentially overlapping expression domains are multi-coloured. A) ANTP-class Hox genes *Sof-Hox3* (dark green), *Sof-Gbx* (orange), and Sof-Mnx* (pink). B) PRD-class genes *Sof-Arx* (red), *Sof-Vsx* (dark blue), Sof-Phox2* (light green), and *Sof-otx*° (yellow). C) LIM-class gene *Sof-Lhx3/4* (light blue). Note that ANTP-class genes are largely restricted to the subesophageal masses, whereas PRD-class genes are more expansive within the supraesophageal mass. Both *Sof-Hox3* and *Sof-Lhx3/4* show extensive expression throughout the nervous system, and five of the eight genes represented here are found within the stellate ganglion. Abbreviations are as in Figure 1; * data extrapolated from Nomaksteinsky *et al*. [26] for stage E29 embryos; ° data extrapolated from Buresi *et al.* [25] for embryos at stages E25–26.

Homeodomain proteins are most well-known for their role in patterning along the anterior-posterior axis, and a large number of these genes are involved in the development of neural tissues in a wide-range of animal lineages. Here we isolate a number of homeobox genes corresponding to the ANTP- PRD- and LIM-classes of HD proteins, profile their expression patterns during the latter half of cephalopod embryogenesis, and report well-defined expression territories that reflect the partitioning of the CNS into regions with distinct gene signatures (Figure 6). Temporal expression profiles of eight HD genes show variable relative expression levels within whole embryos, although analyses of spatial expression patterns of five of these genes reveal constant expression patterns from stages E24-30 – the period of embryogenesis after the establishment of the organ primordia when large-scale tissue differentiation is occurring [14]. Interestingly, we find that LIM and PRD-class genes show similar temporal expression profiles that peak within the whole embryo between stages E25–26, and have spatial expression domains restricted to the central nervous system. In contrast, ANTP-class genes show much broader temporal and spatial expression profiles. ANTP-class genes are well known for their role in early embryo patterning in other animals systems, and thus is not surprising to find these genes active in early cephalopod embryogenesis. The oscillatory nature of ANTP-class temporal gene expression between stages E15–30 suggests at least two waves of up regulation during embryogenesis. We did not examine the spatial expression patterns in embryos younger than stage E24, therefore we cannot confirm whether or not the ANTP- genes are activated in different domains corresponding to the observed early time-point peaks in our temporal analysis, however similar to the PRD- and LIM- class genes, we find ANTP-class gene expression within the CNS during the latter half of embryogenesis.

The central nervous system (CNS) of the adult cuttlefish derives from the fusion of paired ganglia that arise as placodes during the disc phase of development [24]. Neural ganglia are concentrated and fuse medially to form the lobes of the adult CNS when the embryonic disc elevates from the yolk (Stages E21–22; [15]) (see Figure 1). Although extensive comparative data is limited pertaining to PRD-class gene expression in cephalopods, available data suggests that this gene class is largely restricted to the SPM – and thus to the anterior-most portion of the CNS. Aside from transient expression of *So-otx* in the pedal ganglia at stages E20–22 [25], expression of only one other PRD-class HD gene has been reported within the SM: Nomaksteinsky *et al.* [26] report expression at stage E29 of an ortholog of the PRD-class gene family Phox2 (*So-Phox2*) within cells of the posterior-most portion of the aSM and the pSM, as well as within the stellate ganglia (SG). All other PRD-class HD genes appear to be restricted to the more anterior portion of the CNS, within the SPM and optic lobes: the cuttlefish PRD-class gene *Sof-Arx* expresses strongly within the SPM at the anterior-most region (the adult frontal lobes: this paper), *Sof-Vsx* shows expression throughout the optic lobes and within the dorsal basal lobe of the SPM (this paper), the orthodenticle gene (*So-otx*) is expressed within the anterior portion of the CNS (SPM and OL) from stages E20 through hatching [25]. Particularities of these individual gene profiles are addressed below.

The SPM contains many areas that are silent in response to electrical stimulation, presumably cognitive centers, and the lobes that form from the anterior/dorsal portion of the SPM (frontal and vertical lobes) have been implicated in learning and memory [27]. Aristaless-like (Arx) orthologs play an essential role in the patterning of the vertebrate forebrain [28], and are known to be involved in neural migration and specification of GABAergic neurons in worms and vertebrates [29] [30] [31]. *Sof-Arx* is expressed within a well-defined territory in the anterior SPM, homologous to the cerebral ganglia in gastropod mollusks, suggesting a conserved role in anterior CNS patterning. There is currently no data available with regards to GABAergic cell populations in the cuttlefish, however in the octopus *Eledone cirrhosa* GABAergic cells are found within the aSM and mSM as well as within the SPM [32], and Norekian *et al.* [33] report the presence of a GABAergic cell population within the cerebral ganglion of the gastropod mollusk *Clione limancina*. Together these data suggest a homologous GABAergic cell population of in the SPM of the cuttlefish that could be regulated by *Sof-Arx*.

Whereas in vertebrates *Vsx* expression the in visual system has been well documented, expression data for visual system homeobox (*Vsx*) orthologs from non-vertebrate lineages is limited to three model systems: the fruitfly *Drosophila malanogaster* expresses *Vsx* in the optic-lobe progenitor cells [34], the nematode roundworm *Ceanorhabditis elegans* expresses *Vsx* within a small number of sensory interneurons neurons [35], and the ascidian *Ciona intestinalis* where expression in the tadpole is limited to four cells within the visceral ganglion [36]. Expression throughout the optic lobes of the *Sof-Vsx* is unsurprising as it is assumed that this lobe is used for processing of visual information. These lobes also express *Sof-Lhx3/4* (this paper) and the PDR-class gene *so-pax6* [15], and all three of these genes have been proposed as a common molecular signature for a homologous cell type involved in visual processing [37]. We do not comment on possible expression in the retina of any of the genes examined here due to high levels of non-specific background staining regularly found associated with the eye (data not shown). Vsx genes are also known to be expressed in populations of interneurons in both deuterostomes (*Ciona* and vertebrates including the hind brain and spinal cord in zebrafish: [38]), and protostomes (*C. elegans* [35]), and have been proposed to play an ancestral role in integration of sensory information in general [35]. In the cuttlefish, the dorsal basal lobe innervates the optic glands, and lesions in this area of the adult brain show no detectable motor effects [39]. The presence of a *Sof-Vsx* expression domain in this lobe suggests involvement in the specification of a neuronal sub-type involved in the integration of visual cues. Expression of *Sof-Vsx* in cells of the buccal mass and mSM is more intriguing, and may indicate the presence of sensory tissues associated with the buccal mass, whereas expression within the mSM in similar domains as described for *Sof-Lhx3/4* could indicate a concentration of interneurons in this lobe, as both genes are known to be involved in interneuron development. [35], [38], [40].

Whereas the lobes of the SPM are predominantly cognitive centers, the SM is largely composed of motor centers [39]. Lhx3/4 forms part of a motor neuron molecular signature (together with Mnx) [26]. The combination of Lhx3/4 and islet, another LIM-class homeodomain protein, is necessary for motor neuron specification in *Drosophila* [11] and vertebrates (chick: [10]; zebrafish: [8]). No data is currently available regarding islet expression in cephalopods, however analysis of chromatophore motor neuron distribution reveals the SM, in particular the pSM, as a sight of concentrated motor neurons [41] [42], confirming earlier reports of motor activity in response to electrical stimulation in these lobes [39]. Here we find that *Sof-Lhx3-4* is broadly expressed throughout the nervous system of the cuttlefish, including the anterior-most portion of the mSM and aSM, potentially overlapping with *so-Mnx* expression domains (Figure 6, [26]) and thus suggesting the presence of a conserved motor neuron molecular signature in cephalopods. The expression profile of one other LIM-class gene, an ortholog of the Apterous/Lhx2/9 paralogy group, has also been reported from *E. scolopes* (*Es-ap*: [43]). *Es-ap* is expressed within the neural tracts of the arms and the optic lobes, similar to *Sof-Lhx3/4* reported here, as well as within the SPM, in broader domains with respect to *Sof-Lhx3/4* in the cuttlefish. Together, these data suggest conservation of a LIM code [9] for neuronal sub-type cell specification within cephalopods.

In contrast to LIM and PRD-class genes, ANTP-genes show broader temporal expression profiles (see Figure 2b), and are largely restricted to the subeosphageal mass (the posterior CNS): *Sof-Hox3* is expressed within the pSM (this paper), *Sof-Gbx* is expressed within the posterior regions of the pSM and mSM (this paper), and *Sof-Msx* is expressed within the anterior mSM and aSM [26]. Hox gene expression has been previously reported in whole mount *in situ* preparations for the Hawaiian bobtail squid *Euprymna scolopes*, another Cephalopod Mollusk [44]. The authors demonstrate dynamic Hox gene expression patterns, and suggest conservation of expression collinearity within the CNS along the anterior-posterior axis as well as novel non-collinear expression domains within the peripheral nervous system. Hox3 orthologs are commonly expressed within the derivatives of the pedal ganglion (the pSM) in both cephalopod species, as well as in the abalone *Haliotis asinina* [45]. Interestingly, *Sof-Hox3* is also expressed within a small number of cells in the inferior frontal lobe of the SPM (derivative of the cerebral ganglion) in the cuttlefish, whereas no Hox expression was reported within the SPM of *E. scolopes* [44]. The more diffuse expression of *Sof-Hox3* within the pSM and mSM reported here may reflect a similar role in axial patterning of the CNS as described for *E. scolopes*, whereas the well-defined expression domain within the dorsal-posterior pSM and the few cells of the SPM likely reflects the use of *Sof-Hox3* as part of the molecular signature of specific cell types involved in the functional partitioning of this lobe as opposed to conferring positional axial information.

The expression territory of the ANTP-class gastrulation brain homeobox (*gbx*) homolog is consistent with a role in axial patterning of the nervous system. In vertebrates the mid-brain/hind-brain boundary is specified by two HD transcription factors, *gbx* and the PRD-class gene orthodenticle (*otx*) [46]. *Platynereis dumerilii* (polychaete) orthologs are used in a similar manner along the anterior-posterior axis [47]. The cuttlefish orthodenticle gene (*So-otx*) is expressed within the anterior portion of the CNS (SPM and OL) from stages E20 through hatching [25], whereas we show here that *Sof-Gbx* is expressed more posteriorly in the pSM and mSM. Taken together, these data demonstrate that otx/gbx patterning of the A-P axis of the nervous system may be conserved in the cuttlefish. Restriction of expression to the ventral-most regions of the SM however, suggests a role in dorsal/ventral patterning, and nonetheless helps define a specific gene signature for neurons of the magnocellular lobe (ventral-most portion of the SM).

Hirth and Reichert [48] have proposed a general scheme wherein global neural patterning with regards to the body axis can be defined by HD gene expression: ANTP-class genes defining the posterior, and PRD-class genes of the orthodentical group defining the anterior portion of metazoan central nervous systems. Our data certainly conforms to this hypothesis: ANT-class HD gene expression is largely restricted to the posterior derivatives of the CNS, the subesophageal mass, conferring spatial information in the form of broad expression domains along the A/P axis; PRD-class HD gene expression is more prevalent in the anterior CNS, the supraesophageal mass. On the other hand, studies of HD gene expression in the cniderian *Hydra* have lead to the proposal that neural specification may precede axial patterning in terms of ancestral HD gene function [49], and thus axial patterning and cell type specification may be considered distinct genetic modules during development. Evidence is accumulating that strongly suggests a role for ANTP-class genes in specifying post-mitotic neuron sub-types, in both vertebrate and invertebrate lineages (reviewed in [50]). The restricted, mostly non-overlapping, expression territories of each individual HD gene described here reflects the functional partitioning of the adult CNS, and illustrates that the individual lobes of the brain possess a unique molecular signature in terms of HD gene expression. Although cephalopods are not widely used as experimental genetic models, they have a large, well characterized, behavioral repertoire (reviewed in [51]). As our understanding of the genetic architecture underlying the functional differentiation of their nervous systems is unraveled, the cephalopod research community is well placed to make rapid progress in connecting behavioral circuitry with the underlying genetic components.

## Supporting Information

Figure S1 **Phylogenetic analysis of recovered homeodomain gene fragments.** *Sepia* sequences recovered in this study are indicated in boldface type. All major gene clades are indicated, and the *Sepia* sequences resolve together with other mollusk sequences where available. (*) indicates elongation of already known gene sequences. (**) indicates new sequence data. Genes for which expression data is presented here are highlighted. Sequences without a GenBank accession number listed were retrieved from the HomeoDB database (http://homeodb.cbi.pku.edu.cn/: [1] [2]).
(TIF)

Figure S2 **Effect of Adobe Photoshop image manipulation used in this work.** On the left is an original image taken at the microscope showing the background coloration on a scale from pink to green; on the right is the adjusted image after HIGHPASS Filter with a 250 pixel radius and adjustment of brightness and contrast. The background colouration results in an even grey scale after this procedure. Circles represent the areas where background colour was sampled from both images.
(TIFF)

Supporting Information S1 **Homeodomain multiple sequence alignment.** a) CLUSTAL O (1.2.0) multiple sequence alignment used for phylogenetic analysis. Sequences recovered in the current study are highlighted in boldface type. Human, Amphioxus, and Fruitfly sequences were downloaded from the HomeoDB website (http://homeodb.cbi.pku.edu.cn [1] [2]); for all other sequences the genbank accession number is provided. b) Novel short homeodomain nucleotide sequences recovered in the current study.
(DOC)
