# Supplementary figures and images for: Characterization of Homeobox Genes Reveals Sophisticated Regionalization of the Central Nervous System in the European Cuttlefish Sepia officinalis
Source: PLoS One. 2014 Oct 6;9(10):e109627. doi: 10.1371/journal.pone.0109627 (PMC4186843; doi:10.1371/journal.pone.0109627)

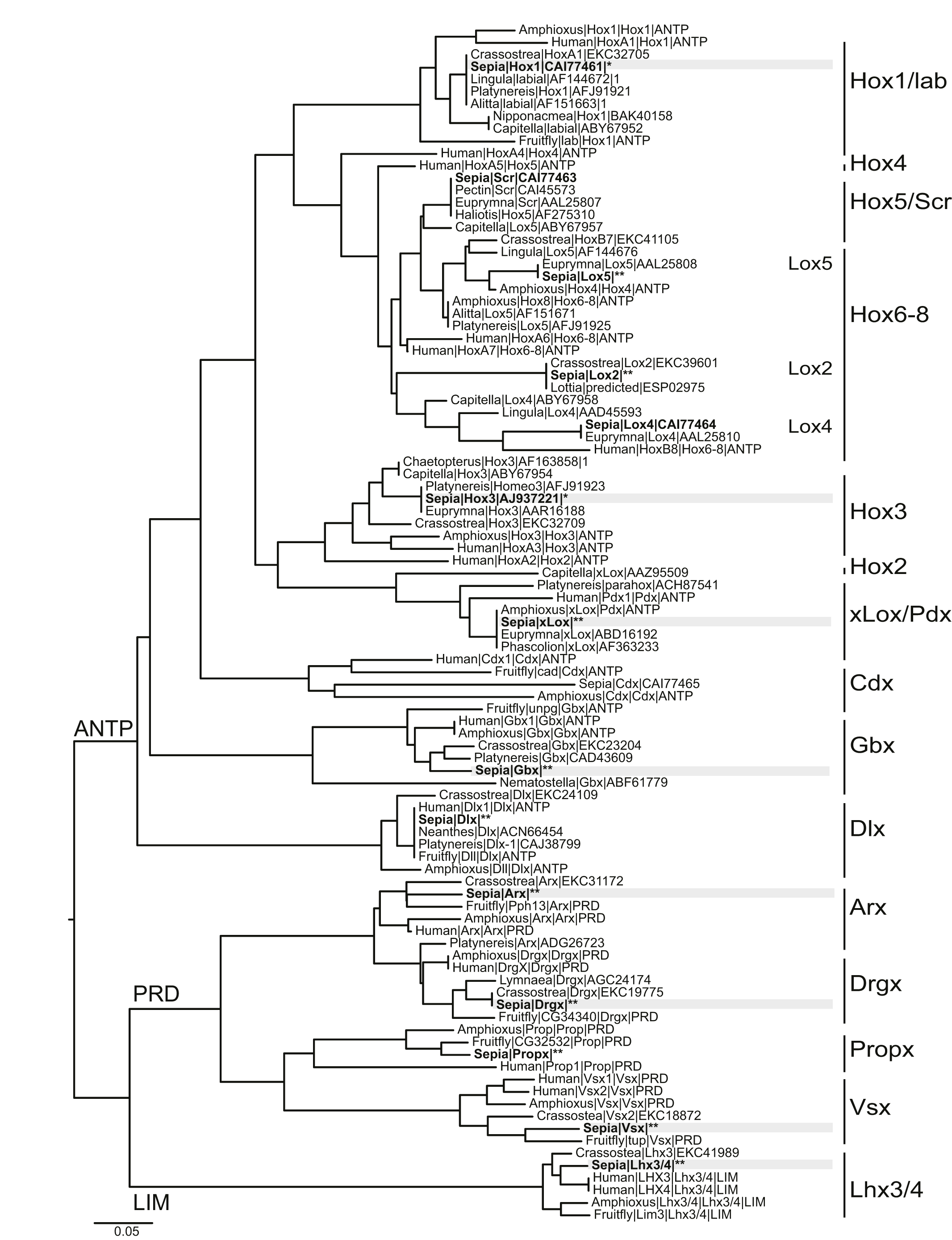

Supplement: Figure S1 — Phylogenetic analysis of recovered homeodomain gene fragments. Sepia sequences recovered in this study are indicated in boldface type. All major gene clades are indicated, and the Sepia sequences resolve together with other mollusk sequences where available. (*) indicates elongation of already known gene sequences. (**) indicates new sequence data. Genes for which expression data is presented here are highlighted. Sequences without a GenBank accession number listed were retrieved from the HomeoDB database (http://homeodb.cbi.pku.edu.cn/: [1] [2]). (TIF) [file pone.0109627.s001.tif]

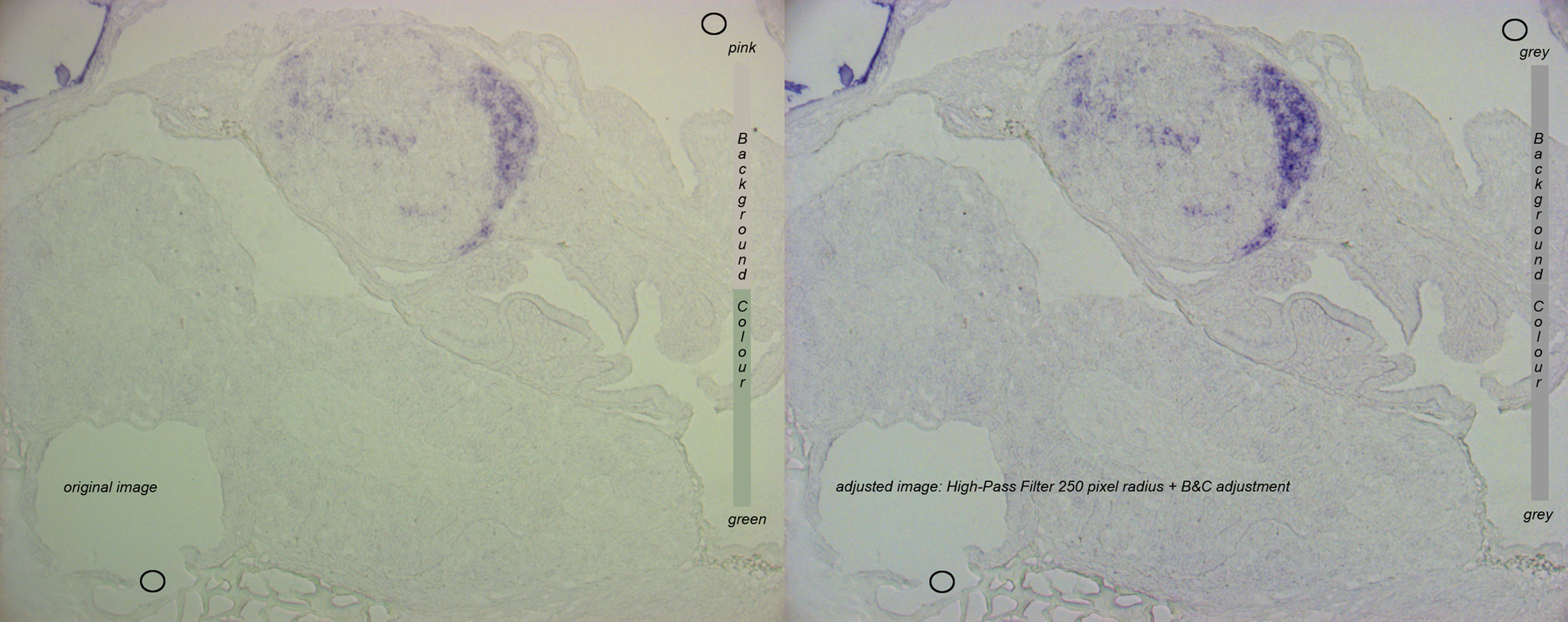

Supplement: Figure S2 — Effect of Adobe Photoshop image manipulation used in this work. On the left is an original image taken at the microscope showing the background coloration on a scale from pink to green; on the right is the adjusted image after HIGHPASS Filter with a 250 pixel radius and adjustment of brightness and contrast. The background colouration results in an even grey scale after this procedure. Circles represent the areas where background colour was sampled from both images. (TIFF) [file pone.0109627.s002.tiff]
