# Supplementary material for: Characterization of Homeobox Genes Reveals Sophisticated Regionalization of the Central Nervous System in the European Cuttlefish Sepia officinalis
Source: PLoS One. 2014 Oct 6;9(10):e109627. doi: 10.1371/journal.pone.0109627 (PMC4186843; doi:10.1371/journal.pone.0109627)
Supplement: Supporting Information S1 — Homeodomain multiple sequence alignment. a) CLUSTAL O (1.2.0) multiple sequence alignment used for phylogenetic analysis. Sequences recovered in the current study are highlighted in boldface type. Human, Amphioxus, and Fruitfly sequences were downloaded from the HomeoDB website (http://homeodb.cbi.pku.edu.cn [1] [2]); for all other sequences the genbank accession number is provided. b) Novel short homeodomain nucleotide sequences recovered in the current study. (DOC) [file pone.0109627.s003.doc]

**Supporting information S1a**. **CLUSTAL O(1.2.0) multiple sequence alignment used for phylogenetic analysis.** Sequences recovered in the current study are highlighted in boldface type. Human, Amphioxus, and Fruitfly sequences were downloaded from the HomeoDB website (http://homeodb.cbi.pku.edu.cn); for all other sequences the genbank accession number is provided.

Human|Cdx1|Cdx|ANTP EFHYSRYITIRRKSELAANLGLTERQVK

**Sepia|Lox4|CAI77464| EFQYNNYLTRKRRIEVAHALNLSERQVK**

Euprymna|Lox4|AAL25810 EFQYNNYLTRKRRIEVAHALNLSERQV-

Human|HoxB8|Hox6-8|ANTP EFLFNPYLTRKRRIEVSHALGLTERQVK

Lingula|Lox4|AAD45593 EFQFNHYLTRKRRIEVAHALCLTERQIK

Capitella|Lox4|ABY67958 EFQFNHYLTRKRRIEIAHALCLTERQIK

Amphioxus|Hox8|Hox6-8|ANTP EFHFNKYLTRRRRIEIAHALGLTERQIK

Euprymna|Lox5|AAL25808 efhfnryltrrrrieiahslglserqik

**Sepia|Lox5|** EFHFNRYLTRRRRIEIAHSLGLSERQIK**

Amphioxus|Hox4|Hox4|ANTP EFHFNRYLTRRRRIEIAHSLGLTERQIK

Crassostrea|HoxB7|EKC41105 EFHFNRYLTRRRRIEIAHLLGLTERQIK

**Sepia|Lox2|** EFKFNRYLTRRRRIELSHMLCLTERQIK**

Lottia|predicted|ESP02975 EFKFNRYLTRRRRIELSHMLCLTERQIK

Crassostrea|Lox2|EKC39601 EFKFNRYLTRRRRIELSHMLCLTERQIK

**Sepia|Scr|CAI77463| EFHYNKYLTRRRRIEIAHALNLTERQIK**

Pectin|Scr|CAI45573 -FHYNKYLTRRRRIEIAHALNLTERQIK

Euprymna|Scr|AAL25807 EFHYNKYLTRRRRIEIAHALNLTERQIK

Haliotis|Hox5|AF275310 EFHYNKYLTRRRRIEIAHALNLTERQIK

Human|HoxA5|Hox5|ANTP EFHFNRYLTRRRRIEIAHALCLSERQIK

Human|HoxA4|Hox4|ANTP EFHFNRYLTRRRRIEIAHTLCLSERQVK

Capitella|Lox5|ABY67957 EFHYNRYLTRRRRIEIAHALQLTERQIK

Lingula|Lox5|AF144676 EFHYNRYLTRRRRIEIAHHLGLTERQIK

Alitta|Lox5|AF151671 EFHYNRYLTRRRRIEIAHALGLTERQIK

Platynereis|Lox5|AFJ91925 EFHYNRYLTRRRRIEIAHALGLTERQIK

Human|HoxA6|Hox6-8|ANTP EFHFNRYLTRRRRIEIANALCLTERQIK

Human|HoxA7|Hox6-8|ANTP EFHFNRYLTRRRRIEIAHALCLTERQIK

Fruitfly|lab|Hox1|ANTP EFHFNRYLTRARRIEIANTLQLNETQVK

Amphioxus|Hox1|Hox1|ANTP EFHYNKYLTRARRVEIAAALNLNETQVK

Human|HoxA1|Hox1|ANTP EFHFNKYLTRARRVEIAASLQLNETQVK

**Sepia|Hox1|CAI77461|* EFHFNKYLTRARRIEIAAALGLNETQVK**

Lingula|labial|AF144672|1 EFHFNKYLTRARRIEIAAALGLNETQVK

Platynereis|Hox1|AFJ91921 EFHFNKYLTRARRIEIAAALGLNETQVK

Alitta|labial|AF151663|1 EFHFNKYLTRARRIEIAAALGLNETQVK

Crassostrea|HoxA1|EKC32705 EFHFNKYLTRARRIEIAAALGLNETQVK

Nipponacmea|Hox1|BAK40158 EFHFNKYLTRARRIEIAASLGLNETQVK

Capitella|labial|ABY67952 EFHFNKYLTRARRIEIAASLGLNETQVK

Human|HoxA2|Hox2|ANTP EFHFNKYLCRPRRVEIAALLDLTERQVK

Crassostrea|Hox3|EKC32709 EFHFNRYLCRPRRIEMAALLSLTERQIK

Chaetopterus|Hox3|AF163858|1 EFHFNRYLCRPRRIEMAALLNLTERQIK

Capitella|Hox3|ABY67954 EFHFNRYLCRPRRIEMAALLNLTERQIK

**Sepia|Hox3|AJ937221|* EFHFNQYLCRPRRIEMAALLNLSERQIK**

Euprymna|Hox3|AAR16188 EFHFNQYLCRPRRIEMAALLNLSERQIK

Platynereis|Homeo3|AFJ91923 EFHFNRYLCRPRRIEMAALLNLSERQIK

Amphioxus|Hox3|Hox3|ANTP EFHFNRYLCRPRRVEMAAMLNLTERQIK

Human|HoxA3|Hox3|ANTP EFHFNRYLCRPRRVEMANLLNLTERQIK

Capitella|xLox|AAZ95509 EFHFNRYITRPRRVELAAHLNLTEQHIK

Human|Pdx1|Pdx|ANTP EFLFNKYISRPRRVELAVMLNLTERHIK

Amphioxus|xLox|Pdx|ANTP EFHFNKYISRPRRIELAAMLNLTERHIK

**Sepia|xLox|** EFHFNKYISRPRRIELAAMLNLTERHIK**

Euprymna|xLox|ABD16192 EFHFNKYISRPRRIELAAMLNLTERHIK

Phascolion|xLox|AF363233 EFHFNKYISRPRRIELAAMLNLTERHIK

Platynereis|parahox|ACH87541 EFHFNKYISRPRRIELASMLSLTERHIK

**Sepia|Lhx3/4|** AYNESPKPARHVREQLSVETGLDMRVVQ**

Crassostea|Lhx3|EKC41989 AYNESPKPARHVREQLSAETGLDMRVVQ

Human|LHX3|Lhx3/4|LIM AYNTSPKPARHVREQLSSETGLDMRVVQ

Human|LHX4|Lhx3/4|LIM AYKNSPKPARHVREQLSSETGLDMRVVQ

Amphioxus|Lhx3/4|Lhx3/4|LIM AYQNSPKPARHVREQLSQETGLDMRVVQ

Fruitfly|Lim3|Lhx3/4|LIM AYNNSPKPARHVREQLSQDTGLDMRVVQ

Amphioxus|Drgx|Drgx|PRD VFAQTHYPDVFTREELAMKINLTEARVQ

Human|DrgX|Drgx|PRD VFAQTHYPDVFTREELAMKINLTEARVQ

Lymnaea|Drgx|AGC24174 AFAQTHYPDVFMREDLAMRINLTEARVQ

Fruitfly|CG34340|Drgx|PRD AFAQTHYPDVFTREDLAMKINLTEARVQ

**Sepia|Drgx|** AFAQTHYPDVFTREDLAMRINLTEARVQ**

Crassostrea|Drgx|EKC19775 AFAQTHYPDVFTREDLAMRINLTEARVQ

**Sepia|Arx|** AFHKTHYPDVFCREELALRIDLTEARVQ**

Fruitfly|Pph13|Arx|PRD AFQRTHYPDVFFREELAVRIDLTEARVQ

Platynereis|Arx|ADG26723 AFQKTHYPDVFTREELAMRINLTEARVQ

Crassostrea|Arx|EKC31172 AFQKTHYPDVFMREELAMRIDLTEARVQ

Amphioxus|Arx|Arx|PRD AFAKTHYPDVFTREELAMRVDLTEARVQ

Human|Arx|Arx|PRD AFQKTHYPDVFTREELAMRLDLTEARVQ

Human|Vsx1|Vsx|PRD AFSEAHYPDVYAREMLAVKTELPEDRIQ

Amphioxus|Vsx|Vsx|PRD AFNEAHYPDVYAREMLAMKTDLPEDRIQ

Human|Vsx2|Vsx|PRD AFNEAHYPDVYAREMLAMKTELPEDRIQ

Crassostea|Vsx2|EKC18872 AFKDAHYPDVYAREVLALKTSLPEDRIQ

Fruitfly|tup|Vsx|PRD AFKEAHYPDVYAREMLSLKTELPEDRIQ

**Sepia|Vsx|** AFKEAHYPDVYAREVLSLKTDLPEDRIQ**

Amphioxus|Prop|Prop|PRD AFSKSHYPDIYVREELARATKLNEARIQ

Fruitfly|CG32532|Prop|PRD AFAKSHYPDIYCREELARTTKLNEARIQ

**Sepia|Propx|** AFAKSHYPDIYCREELARITKLNEARIQ**

Human|Prop1|Prop|PRD AFGRNQYPDIWARESLARDTGLSEARIQ

Sepia|Cdx|CAI77465 --HYNLYINVIRKAELAQELGLSQRQIK

Amphioxus|Cdx|Cdx|ANTP EFYSNKYITIKRKVQLANELGLSERQVK

Fruitfly|cad|Cdx|ANTP EYCTSRYITIRRKSELAQTLSLSERQVK

Crassostrea|Dlx|EKC24109 RFQRTQYLALPERAELAASLGLTQTQPL

Amphioxus|Dll|Dlx|ANTP RFQRTQYLALPERAELAAQLGLTQTQVK

Human|Dlx1|Dlx|ANTP RFQQTQYLALPERAELAASLGLTQTQVK

**Sepia|Dlx|** ------YLALPERAELAASLGLTQTQVK**

Neanthes|Dlx|ACN66454 RFQRTQYLALPERAELAASLGLTQTQVK

Platynereis|Dlx-1|CAJ38799 RFQRTQYLALPERAELAASLGLTQTQVK

Fruitfly|Dll|Dlx|ANTP RFQRTQYLALPERAELAASLGLTQTQVK

Nematostella|Gbx|ABF61779 EFHNKKYVSLEERSVIATNLNLTEVQVK

Fruitfly|unpg|Gbx|ANTP EFHAKKYLSLTERSQIATSLKLSEVQVK

Human|Gbx1|Gbx|ANTP EFHCKKYLSLTERSQIAHALKLSEVQVK

Crassostrea|Gbx|EKC23204 EFHSKKYLSLTERSHIAHNLKLSEVQVK

**Sepia|Gbx|** EFHSKKYLSLIERSQIAHNLKLSEVQVK**

Amphioxus|Gbx|Gbx|ANTP EFHSKKYLSLTERSQIAHALKLSEVQVK

Platynereis|Gbx|CAD43609 EFHSKKYLSLTERSQIAHNLKLSEVQVK

**Supporting information S1b. Recovered novel short homeodomain sequences.**

>Sof-Dlx ([Sepia officinalis] distalless homeotic protein mRNA, partial CDS

TTTGGCCTTACCCGAGCGAGCCGAGTTGGCCGCTTCTCTTGGCCTCACACAAACCCAGGTGAAAATA

>Sof-Dlx ([Sepia officinalis] distalless-like homeotic protein translated mRNA, partial CDS

YLALPERAELAASLGLTQTQVKI

>Sof-Lox5 [Sepia officinalis] Homeodomain protein similar to Lox4 mRNA, partial cds

TCCATTTTAATCGATACCTCACCCGCAGACGGCGGATAGAAATCGCTCATTCCTTGGGACTATCTGAGAGACAAATAAAA

>Sof-Lox5 [Sepia officinalis] Homeodomain protein similar to Lox4 translated mRNA, partial cds

EFHFNRYLTRRRRIEIAHSLGLSERQIK

>Sof-Lox2 [Sepia officinalis] Homeodomain protein similar to Lox2 mRNA, partial cds.

TTAAGTTTAACCGGTATCTCACGCGGCGCCGACGTATTGAGCTTTCGCATATGCTCTGTTTGACGGAACGACAAATAAAA

>Sof-Lox2 [Sepia officinalis] Homeodomain protein similar to Lox2 translated mRNA, partial cds

EFKFNRYLTRRRRIELSHMLCLTERQIK

>Sof-Propx [Sepia officinalis] Homeodomain protein similar to PROP mRNA, partial cds

GCTGCCTTCGCCAAAAGCCACTACCCTGACATATACTGCCGGGAGGAACTCGCCCGGATCACGAAACTCAACGAGGCACGCATACAGGTTT

>Sof-Propx [Sepia officinalis] Homeodomain protein similar to PROP translated mRNA, partial cds

AAFAKSHYPDIYCREELARITKLNEARIQVW
